# Is an SV caller compatible with sequencing data? An online recommendation tool to automatically recommend the optimal caller based on data features

**DOI:** 10.3389/fgene.2022.1096797

**Published:** 2023-01-06

**Authors:** Shenjie Wang, Yuqian Liu, Juan Wang, Xiaoyan Zhu, Yuzhi Shi, Xuwen Wang, Tao Liu, Xiao Xiao, Jiayin Wang

**Affiliations:** ^1^ School of Computer Science and Technology, Xi’an Jiaotong University, Xi’an, China; ^2^ Shaanxi Engineering Research Center of Medical and Health Big Data, Xi’an Jiaotong University, Xi’an, China; ^3^ Annoroad Gene Technology (Beijing) Co. Ltd, Beijing, China; ^4^ Geneplus Shenzhen, Shenzhen, China

**Keywords:** sequencing data analysis, bioinformatics tool, software recommendation, structural variant caller, meta-learning framework

## Abstract

A lot of bioinformatics tools were released to detect structural variants from the sequencing data during the past decade. For a data analyst, a natural question is about the selection of a tool fits for the data. Thus, this study presents an automatic tool recommendation method to facilitate data analysis. The optimal variant calling tool was recommended from a set of state-of-the-art bioinformatics tools by given a sequencing data. This recommendation method was implemented under a meta-learning framework, identifying the relationships between data features and the performance of tools. First, the meta-features were extracted to characterize the sequencing data and meta-targets were identified to pinpoint the optimal caller for the sequencing data. Second, a meta-model was constructed to bridge the meta-features and meta-targets. Finally, the recommendation was made according to the evaluation from the meta-model. A series of experiments were conducted to validate this recommendation method on both the simulated and real sequencing data. The results revealed that different SV callers often fit different sequencing data. The recommendation accuracy averaged more than 80% across all experimental configurations, outperforming the random- and fixed-pick strategy. To further facilitate the research community, we incorporated the recommendation method into an online cloud services for genomic data analysis, which is available at https://c.solargenomics.com/ *via* a simple registration. In addition, the source code and a pre-trained model is available at https://github.com/hello-json/CallerRecommendation for academic usages only.

## 1 Introduction

In genomics and bioinformatics, calling structural variants (SVs) from sequencing data is a somewhat straightforward topic (Handsakeret al., 2011; Northcott et al., 2012; English et al., 2014; Cao et al., 2014; Guan and Sung, 2016; Chiang et al., 2017). Tens of review papers (Seo et al., 2016; Fang et al., 2019; Kosugi et al., 2019; Amarasinghe et al., 2020; Luan et al., 2020; Zhao et al., 2020; Zook et al., 2020; De Coster et al., 2021; Guo et al., 2021) highlight SVs as important biomarkers and routinely identify them in various fields. Therefore, many SV callers have been developed to detect SVs (Stancu et al., 2017; Gong et al., 2018; Sedlazeck et al., 2018; Wenger et al., 2019; Jiang et al., 2020).

These callers used different strategies. Read pairs and depth approaches (Kosugi et al., 2019) primarily use the discordant alignment and depth features of paired-end reads that encompass or overlap an SV. The split read approach (English et al., 2014) primarily uses split alignment features of single- or paired-end reads that span an SV breakpoint. The assembly approach (Seo et al., 2016) detects SVs primarily by aligning assembled contigs with entire or unmapped sequencing reads to the reference sequence.

In summary, different strategies investigate various variant signals (values and/or distributions) in sequencing data and can deal with diverse sequencing data with different signals and their distributions. Furthermore, some empirical studies (Luan al., 2020; Kosugi et al., 2019; Guo et al., 2021) have been conducted to validate this phenomenon. A set of popular callers is compared on some benchmarking datasets in these studies, and the results showed that most callers have an edge for specific data.

In such instances, using the signal distributions in a given sequencing data to select the proper caller for diverse sequencing data makes sense. However, these signal distributions are usually ambiguous. When faced with a practice SV calling problem, it is difficult for users, especially non-experts, to decide which caller to use. Three simple approaches are commonly used in practice. First, choose one at random (random-pick strategy). Second, select one that is familiar or popular (fixed-pick strategy). Finally, consult an expert who will analyze the relationship between the sample and the SV caller’s performance and make a recommendation based on their experience. The first two approaches are straightforward, but their efficacy cannot be guaranteed. The last one can sometimes boost effectiveness. However, there are very few such experts available to meet real-world demands.

Consequently, selecting appropriate SV callers becomes an urgent issue. As different SV callers explore different distributions (or patterns in some approaches) in sequencing data to make decisions, these distributions in sequencing data can affect the caller’s performance. It is logical to use some signal distributions for SV caller selection. Thus, this study proposes an automatic SV caller recommendation method. The SV caller selection problem is established under a meta-learning framework in the method, with calling SVs from the sequencing data as the learning problem and caller selection as the meta-learning problem (Rendell and Cho, 1990; Ilchenkov and Pendryak, 2015; Morais et al., 2016; Sousa et al., 2016; Cruz et al., 2017; Vilalta and Drissi, 2022). The goal is to use meta-learning to improve the performance of the learning problem.

Specifically, the meta-features are collected to reflect the sequencing data’s features, which attempt to reflect the hidden distributions or patterns in the sequencing data. The meta-targets are identified to indicate the most appropriate SV caller for the given data, and a meta-model is then built to mine the relationship between the meta-features and meta-target. When confronted with an SV caller section problem for a given sequencing data, the meta-features of the data are collected and fed into the constructed meta-model, and the meta-model specifies the final decision on the recommended a SV caller.

Since third-generation sequencing is becoming the major sequencing technology for SV detection (Luan al., 2020; Amarasinghe et al., 2020; Zook et al., 2020; Fang et al., 2019), this study focuses on the SV caller recommendation method on the third-generation sequencing data. A series of experiments are conducted on both simulated and real sequencing data to validate the performance of the recommendation. Compared to the random- and fixed-pick strategies, this recommendation method always selects a better caller, with an average recommendation accuracy of more than 80%. To the best of our knowledge, this study is one of the first automatic recommendation methods for bioinformatics tools for analyzing sequencing data. It can accurately recommend the best caller fits for the available sequencing data. This model is thought to be quite valuable for data analysts. To further facilitate the research community, we incorporated the recommendation method into an online cloud services for genomic data analysis, which is available at https://c.solargenomics.com/ *via* a simple registration. In addition, the source code and a pre-trained model is available at https://github.com/hello-json/CallerRecommendation for academic usages only.

## 2 Methods

### 2.1 Overview of the methods

The historical datasets in this specific meta-learning problem are the sequencing datasets with benchmarks, while the new dataset is the sequencing data to be detected. This meta-learning problem mines the potential relationship between meta-features and meta-target (appropriate callers) from sequencing datasets with benchmarks and recommends appropriate callers for the sequencing data to be detected based on this relationship.

**FIGURE 1:**
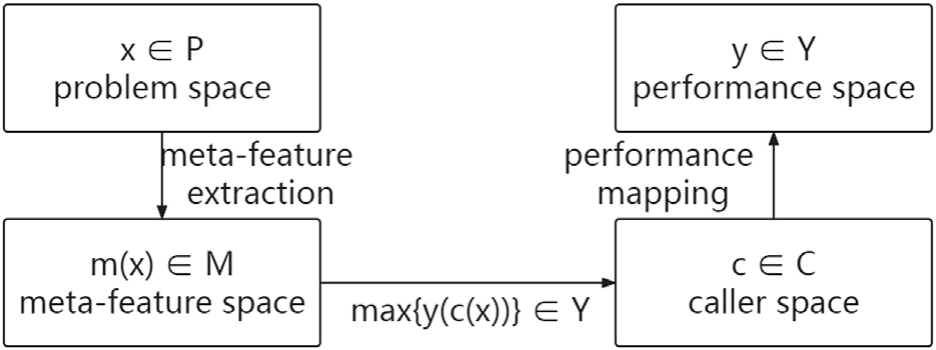
Abstract model of the SV caller selection problem.

A function f is created to map meta-features of the sequencing datasets to appropriate callers. The best function

$$f^{*}={argmin}_{\left\{f\right\}}\left(L\left(f\right)\right)$$ (1)

is obtained by minimizing the loss function L based on caller performance on historical SVs calling problems. L is a function that measures the difference between the recommended and appropriate callers. Thus, the meta-learning function for the SV caller selection problem P can be formalized as follows: find the meta-learning function $f\left(m\left(x\right)\right)$ to the caller space C for a given sequencing data x∈P with meta-features $m\left(x\right)\in M$ . The chosen caller c maximizes the performance mapping $y\left(c\left(x\right)\right)\in Y$ . That is,

$$f\left(m\left(x\right)\right)\to C:\max\left\{y\left(c\left(x\right)\right)\right\}\in Y\left(x\in P,\;m\left(x\right)\in M\right)$$ (2)

where P , M , C , and Y represent the problem space (sequencing dataset), meta-feature space (meta-feature set), caller space (SV caller set), and performance space (caller performance interval), respectively. Furthermore, $m\left(x\right)$ and $c\left(x\right)$ are the meta-features and the appropriate callers of x , respectively. Usually, the most important element is determining which caller outperforms the others. Thus, f can be improved further to map the features of P to the best caller. Figure 1 shows an abstract model of the SV caller selection problem.

**FIGURE 2:**
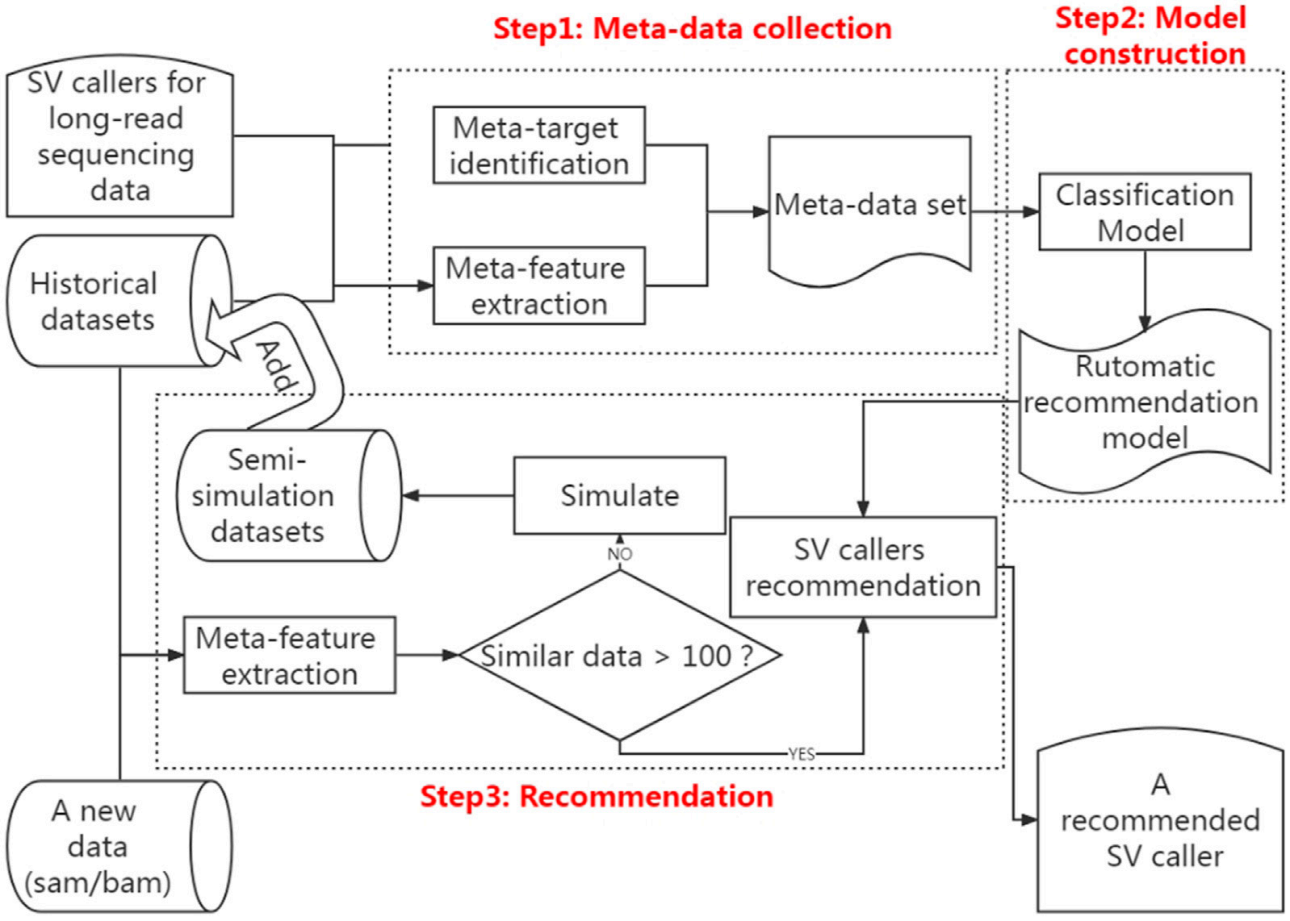
Computational pipeline for automatically recommending the optimal SV caller according to the features of sequencing data.

According to the above analysis, a classification algorithm can build the meta-model in the SV caller recommendation method. This classification algorithm learns the relationship between the meta-features of each sequencing data in historical datasets and the optimal caller and then applies this relationship to map the detected sequencing data to its optimal caller. Consequently, the framework created in this study for the recommendation method is divided into three sections, namely, extracting data features and identifying the optimal caller, modeling the relationship between data features and the optimal caller, and recommending the optimal caller. Figure 2 shows the framework of this method.

### 2.2 Metadata collections

As shown in Figure 2, the first step of the SV caller recommendation method is metadata collection, which is divided into two stages, *i.e.,* meta-feature extraction and meta-target identification.

#### 2.2.1 Meta-feature extraction

The meta-features in this context are those sequence alignment and map (SAM)/binary alignment and map (BAM)files features that can effectively differentiate the performance of SV callers. The SAM/BAM file in sequencing data analysis is a sequence text file that contains the sequencing reads with information aligned to the reference genome. The statistical and information theory-based method is currently the most widely used meta-feature extraction method, which extracts meta-features such as dataset sizes, attribute types, numbers of attributes, mean, and variance (Brazdil et al., 2003; Pise, 2013; Ali et al., 2018; Wang et al., 2019). However, sequencing data is a unique type of data in that a single read or statistical information about reads contains little information, and the set of sequencing reads is mapped to a region that contains the most information. Therefore, this method does not perform well with sequencing data. For example, even if SAM/BAM files are very close in size or even have the same number of reads, the information they contain may be completely different due to the different bases of reads.

According to bioinformatics research, read length, sequencing depth, base quality, mapping quality, and insert size significantly impact caller performance (Kosugi et al., 2019; Wang et al., 2019; Zook et al., 2020; Chen et al., 2021). However, these features are used to call SVs, whereas meta-features are now required to effectively differentiate the performance of SV callers. As a result, there are several useless features here. According to this study’s testing, some features, such as read length and sequencing depth, are useful, while others are not. Furthermore, some review studies have proposed some sequencing data features, such as the size of SVs and the proportion of SVs in tandem repeat regions, which have been shown to differentiate the performance of SV callers (Kosugi et al., 2019; Zook et al., 2020; Guo et al., 2021).

For example, Picky (Gong et al., 2018) uses an assembly approach to produce read alignment by stitching the segments from LAST with a greedy seed-and-extend strategy and can thus handle large SVs by assembling them as distinct contigs. However, when the sequencing depth is low, the assembly junctions are ambiguous, i.e., some of the haplotype sequences (particularly contigs of SV alleles) are missing, which may affect SV calling recall. Sniffles (Sedlazeck al., 2018) uses a split read approach to identify SVs by putative variant scoring using several features based on NGMLR alignment results and thus can identify SVs even when the sequencing depth is low. However, due to the lack of assembly, it is difficult to identify large SVs from ambiguous alignments for Sniffles.

Therefore, based on this study’s experiments and review papers, features that can effectively differentiate the performance of SV callers while eight avoiding over-fittings were ultimately chosen. In this context, these are known as meta-features. They were distributed on the SAM/BAM file levels (datasets levels) and the variant signature levels (instance levels). The average length of reads and the average sequencing depth are SAM/BAM file-level meta-features. The proportion of SVs in tandem repeat regions, the proportion of short SVs (50–200 bp), the proportion of middle SVs (200–1,000 bp), the proportion of large SVs (>1,000 bp), read variant burden (the number of SVs that one read can traverse), and the proportion of regions with high read variant burden are among the variant signature level meta-features. Table 1 summarizes the selected meta-features.

**TABLE 1:** Meta-features extracted to characterize sequencing data.

Level	Meta-features
SAM/BAM file level	Average length of reads
	Average sequencing depth
Variant signature level	Proportion of SVs in tandem repeat regions
	Proportion of short SVs
	Proportion of middle SVs
	Proportion of large SVs
	Read variant burden
	Proportion of regions with high read variant burden

This study creates a new meta-feature extraction method, called the variational signature-based meta-feature estimation algorithm, to extract the above features from the sequencing datasets. This is a fast scanning algorithm. It simply needs to estimate the features described above rather than accurately detect the SVs. Thus, while the proposed algorithm may be slightly inaccurate, it has been experimentally proven to affect caller recommendations. The algorithm can extract information from the SAM/BAM file level and variant signature levels. Meta-features, such as sequencing depth, can be obtained from SAM files using samtools. Meta-features were extracted for variant signatures by setting a sliding window and grabbing softclip reads with breakpoint information. Specifically, the loci with variant signatures and the size of SVs can be estimated as follows:

1) Cluster all the reads from the input SAM/BAM file with softclips.
2) Divide the reads with softclips into two categories based on whether the softclip is at the beginning or at the end of the reads.
3) Determine the variant loci of each category according to the cigar value of each read.
4) Determine the distance between pairs of breakpoints as an estimated value of the size of SVs.

Algorithm 1 presents the pseudocode of meta-feature extraction. The algorithm’s input was the SAM/BAM file F, while the output was the meta-feature, MF. The tandem repeat regions used in this study were annotated in the hg19 annotation file, which can be downloaded from UCSC Genome Browser (http://hgdownload.soe.ucsc.edu/goldenPath/hg19/database/rmsk.txt.gz) (Zook et al., 2016).

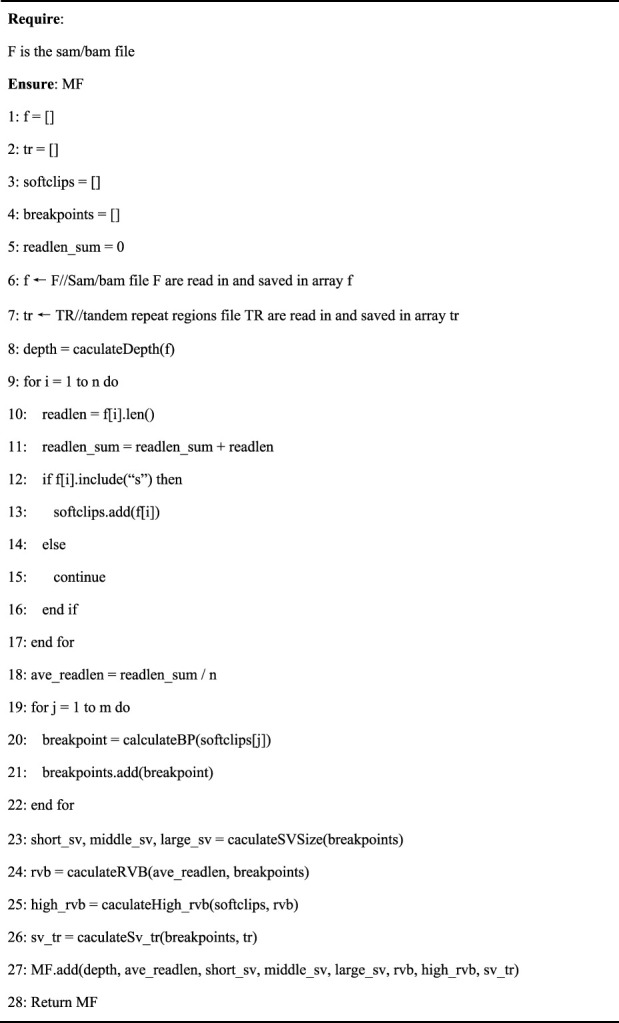

Algorithm 1 Meta-feature extraction

Lines one to six of Algorithm 1 initialize the f, tr, softclips, breakpoint arrays, and readlen_sum. The f array saves the reads in the SAM/BAM file, the tr array saves regions in the tandem repeat regions file, the softclips array saves softclip reads, the breakpoint array saves softclip reads breakpoints, and the readlen_sum is used to save the total length of reads. In pseudocode lines six to seven, SAM/BAM file F and the tandem repeat regions file TR are read in and saved in arrays f and tr, respectively.

In pseudocode line 8, the depth function calculates the sequencing a depth, which is then assigned to depth. The pseudocode lines 9–18 traverse each read length separately, update the readlen_sum value, save softclips in the softclips array, and calculate the average length of softclip reads. The pseudocode lines 19–22 traverse each softclip reads separately, calculate breakpoints, and save them in the breakpoint array. The pseudocode lines 23–27 calculate the short_sv, middle_sv, large_sv, rvb, high_rvb, and sv_tr using the caculateSVSize, caculateRVB, caculateHigh_rvb, and caculateSv_tr functions, respectively, where short_sv, middle_sv, large_sv, rvb, high_rvb, and sv_tr denote the proportion of short SVs, the proportion of middle SVs, the proportion of large SVs, read variant burden, the proportion of regions with high read variant burden, and the proportion of SVs in tandem repeat regions, respectively. Finally, the meta-feature, MF, is saved on line 27 and returned on line 28.

#### 2.2.2 Meta-target identification

This step involves tagging meta-targets representing the best of the callers. In turn, each caller was run on each sequencing data and then ranked based on their performance, and the best was chosen as the meta-target for that data. Algorithm 2 provides the pseudocode of meta-target identification. The algorithm’s inputs include the long-read sequencing dataset as **D**, the set of SV callers as **C**, and the caller performance evaluation metric M. The algorithm’s output is the meta-target set as **T**.

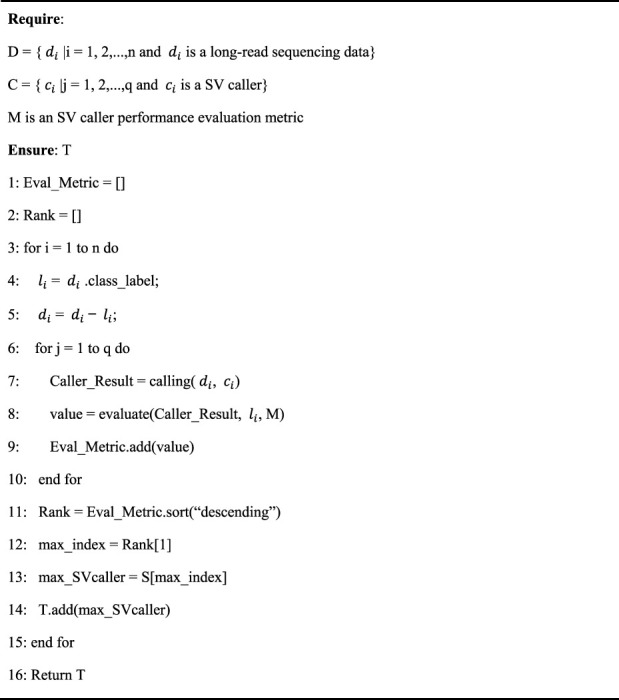

Algorithm 2 Meta-target identification

In Algorithm 2, the Eval_Metric and rank arrays are used in lines 1 and 2. The Eval_Metric array is used to save the performance evaluation values, whereas the rank array is used to save the ranks of callers based on their performance evaluation values. In pseudocode lines 3–15, the meta-target T is identified for each long-read sequencing dataset in ***D***. For a long-read sequencing dataset $d_{i\;}$ , its label is saved in $l_{i}$ and then removed from $d_{i\;}$ lines 4 and 5. In lines 6–10, each caller $c_{i}$ , $d_{i\;}$ is called using the SV caller $c_{i}$ . And the calling results are evaluated in terms of the metric M. The evaluation result is added to Eval_Metric. Eval_Metric is sorted in descending order in line 11. Ranks of SV callers according to their performance in terms of Eval_Metric are saved in rank. The meta-target is then obtained and saved in T in lines 12–14. Finally, the meta-target set T is returned at line 16. The meta-feature and meta-target are saved after the above meta-feature extraction and meta-target identification. Users can specify different meta-targets based on their requirements.

#### 2.2.3 Meta-model construction and recommendation

The features of each data were obtained, and the best caller for that data using the steps outlined above was determined. That is, the meta-features and meta-targets are available. In this case, one meta-feature is a vector $p_{i\;}$ ( ${mf}_{1\;}$ *,* ${mf}_{2\;}$ *,* ${mf}_{3\;}$ *,...* ${mf}_{n\;}$ ), and corresponding to this meta-feature, there is a meta-target, where *i* = 1, 2,..., m, and m is the number of training samples. All samples constitute a dataset that can be used to train a classification model. Therefore, the classifier was used to learn the relationship between the meta-features and meta-targets, and then the meta-model was built as the recommendation model. Finally, the RandomForest algorithm was used to build the classifier after considering the relevance of the features and effectiveness of the model.

This step above results in the automatic recommendation model. When users need to make caller recommendations based on the new long-read sequencing data, they first extract the data’s meta-features from the SAM/BAM file. They then determine whether the number of data in the historical dataset with meta-features similar to the new long-read sequencing data is greater than 100. If not, they generate 100 semi-simulated data based on the meta-features of the real new long-read sequencing data and add them to the historical dataset to retrain the recommendation model. If yes, the extracted meta-features are fed into the recommendation model. Finally, the model will output the recommendation results of the new long-read sequencing data based on the meta-targets that users have specified.

## 3 Results

This section conducts experiments to verify the necessity and efficacy of the proposed recommendation method:

**Question 1. Necessity**: Does the matching degree between the SV caller and the signal distributions significantly impact SV caller performance?

This is an important question. The influence of the matching degree between the detection strategy and the signal distributions determines the necessity of the research on the recommendation method. A fixed or randomly selected SV caller can be used if it exerts minimal influence on the SV caller performance.

**Question 2. Effectiveness**: How effective is the proposed caller recommender?

This is also an important question. Suppose the matching degree between the SV caller and the signal distributions exerts a non-negligible influence on the SV calling performance. In that case, the performance of the proposed SV caller recommendation method determines whether it can be used in practice.

### 3.1 Experiment setup

#### 3.1.1 Benchmark datasets and candidate long-read sequencing data SV callers

The small number of real long-read sequencing datasets with benchmarks that can be analyzed is insufficient to construct a historical dataset. Using the PBSIM simulator, 768 simulated long-read sequencing datasets were generated (Yukiteru al., 2013) (https://github.com/yukiteruono/pbsim2). Specifically, various SVs were planted on chromosome 1, and reads ranging in lengths from 1,000 to 25,000 bps with varying sequencing depths were generated (10–150 X). For each sample, the density of the SVs was varied by varying the distance between the SVs. Furthermore, the proportion of variations in the tandem repeat region was altered by varying the number of SVs within the tandem repeat region of the genome. True called SVs are defined as the called SVs that significantly overlap with the reference SVs by proportions (≥80%).

Five state-of-the-art SV callers, namely, NanoSV (Stancu et al., 2017) (https://github.com/mroosmalen/nanosv), Picky (https://github.com/TheJacksonLaboratory/Picky), Sniffles (https://github.com/fritzsedlazeck/Sniffles), PbSV (Wenger et al., 2019) (https://github.com/PacificBiosciences/pbsv), and CuteSV (Jiang et al., 2020) (https://github.com/tjiangHIT/cuteSV), were implemented on the simulated datasets as the candidate callers. Each of these callers has advantages due to their different strategies. For example, Picky can handle large SVs well by assembling reads as distinct contigs due to the assembly approach it adopts, while the assembly approach performs poorly when the sequencing depth is too low due to the lack of reads. However, due to the split read approach (alignment-based approach), NanoSV, PbSV, Sniffles, and CuteSV can detect SVs even at a low sequencing depth, but they cannot handle large SVs due to lack of read assembling. As another example, Sniffles and CuteSV are appropriate for dense SVs and SVs in repeat-rich regions. Because the sequencing data contain many sequencing errors, particularly for long reads, they have also designed error event filtering mechanisms in their algorithms, which greatly improve the detection of SVs in repeat-rich regions and dense SVs or even nested SVs. However, Picky, NanoSV, and PbSV cannot handle these SVs due to the lack of an error event filtering mechanism. Each caller in the experiments used the default parameters and the alignment tool recommended by the caller developer.

#### 3.1.2 Metrics to evaluate the performance of SV callers

F-measure, precision, and recall are important metrics for evaluating bioinformatics analysis methods, and they are frequently discussed in bioinformatics methodology studies (Kosugi et al., 2019). Therefore, these three metrics were chosen to evaluate the performance of SV callers. Precision, recall, and f-measure are calculated as follows:

$$precision=\frac{TP}{Call}$$ (3)

$$recall=\frac{TP}{Ref}$$ (4)

$$f-measure=\frac{2\times precision\times recall}{precision+recall}$$ (5)

where TP, Call, and Ref are the numbers of true positives, called SVs, and the corresponding reference SVs, respectively.

#### 3.1.3 Evaluating the performance of the recommendation method

The performance of the recommended SV caller is an important evaluation metric (Song et al., 2012). Therefore, recommendation accuracy (RA) is used to evaluate the RA of the proposed recommendation method, reflecting the difference in performance between the recommended optimal SV caller and the real optimal SV caller. During the implementation of the experiments, a leave-one-out cross-validation method was used to calculate RA values.

For a given long-read sequencing data s, let CallerR be the recommended SV caller, CallerO the most optimal SV caller, and CallerW the worst caller. RA is defined as follows:

$$RA\left(s\right)=\frac{P_{CallerR}\left(s\right)-P_{CallerW}\left(s\right)}{P_{CallerO}\left(s\right)-P_{CallerW}\left(s\right)}$$ (6)

Where $P_{X}\left(Y\right)$ denotes the performance of SV caller X on long-read sequencing data Y.

### 3.2 Necessity of the proposed recommendation method

**FIGURE 3:**
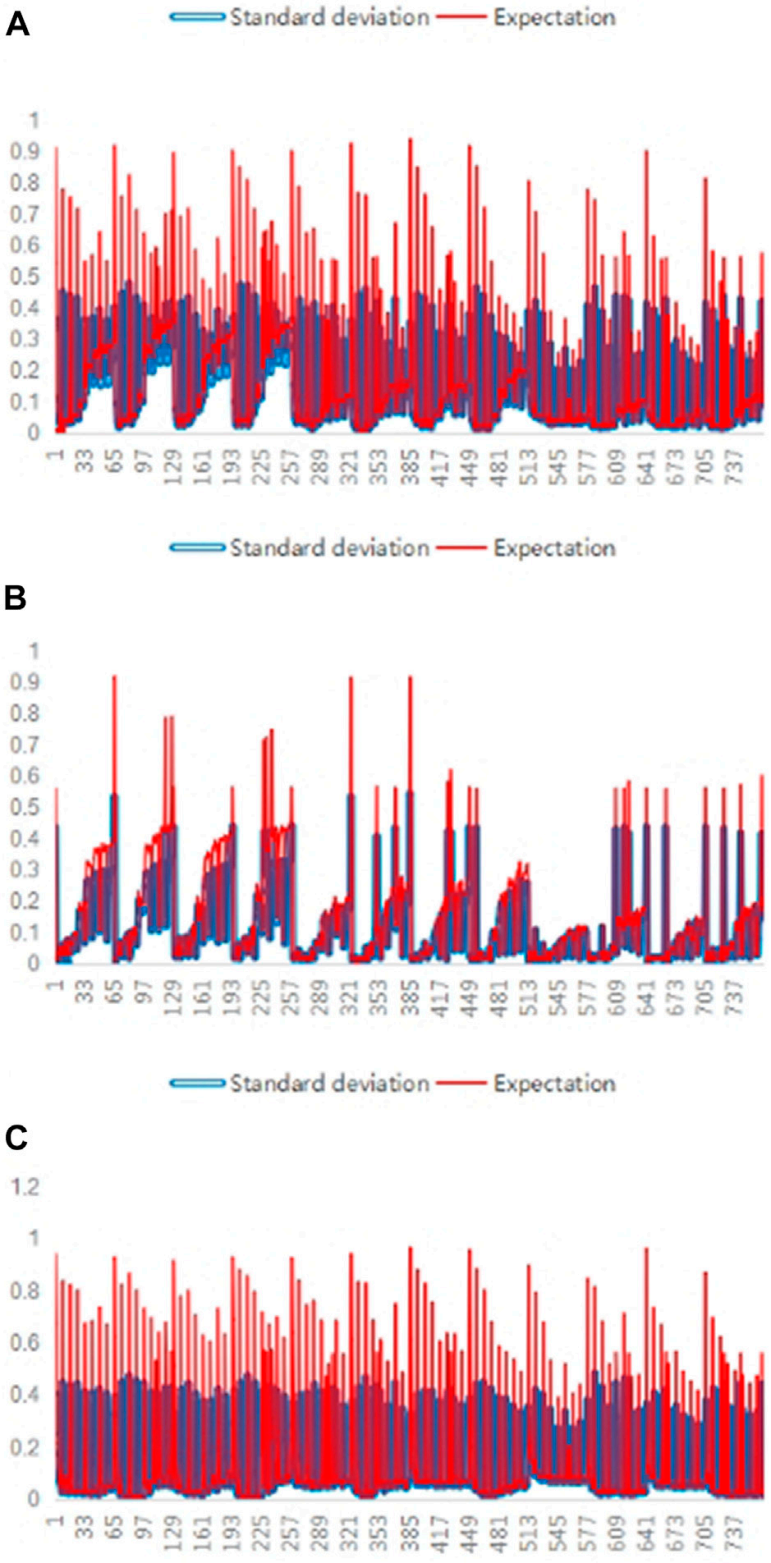
Differences between the various SV callers. In **(A)**, **(B)**, and **(C)**, the values of the three performance evaluation metrics, i.e., f-measure, precision, and recall, were calculated for each of the five callers on 768 long-read sequencing datasets. For each dataset, expectation values for one of the three performance evaluation metrics were calculated and shown with red lines, while the standard deviation values are shown with blue lines in the three subfigures, where the abscissa denotes the number of the long-read sequencing datasets and the ordinate denotes the corresponding performance evaluation metric value.

The f-measure, precision, and recall values of the five SV callers were compared on different long-read sequencing data to evaluate the extent of performance differences between them, as shown in Figure 3.

As shown in Figure 3, the standard deviation values are non-negligible compared to the expected values for any meta-targets, indicating a significant difference in the performances of SV callers.

**FIGURE 4:**
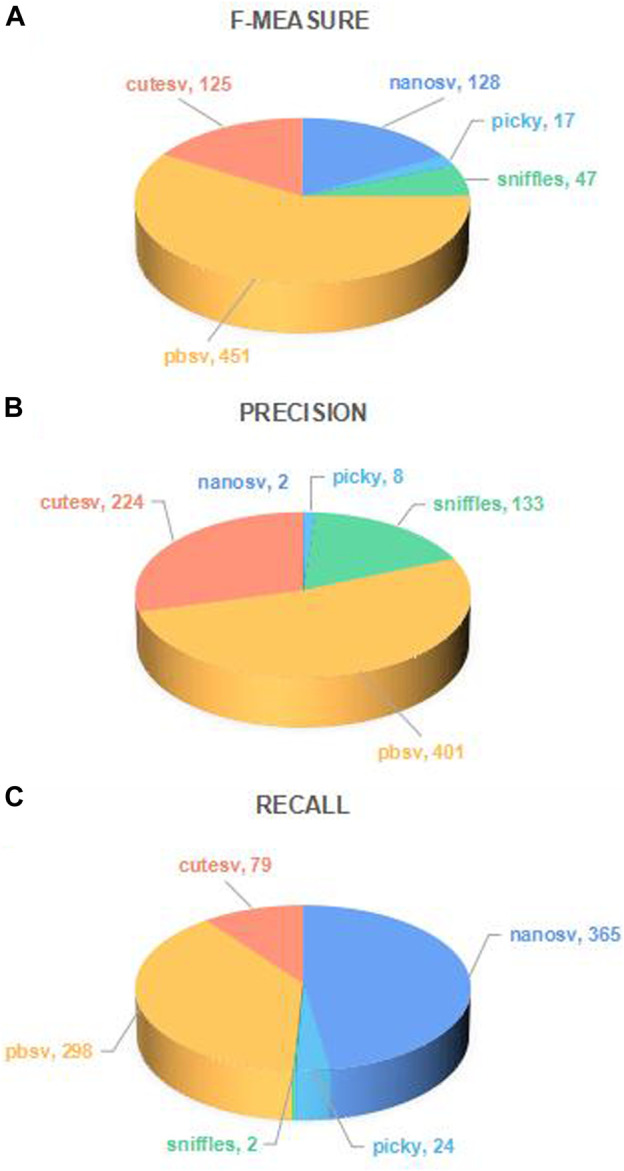
Number of long-read sequencing datasets that each SV caller ranks as top 1. The number of times each caller achieved the top one in the three performance metrics of f-measure, precision, and recall for 768 long-read sequencing datasets was calculated in **(A)**, **(B)**, and **(C)**. In each subfigure, the sectors of different colors represent different callers, and the sector’s size indicates the proportion of different callers achieving the top 1. The number marked in each sector is the number of long-read sequencing datasets on which the performance of the SV caller in terms of the performance evaluation metric value ranks top 1.

Although the performance of SV callers varies significantly, this recommendation method of research is unnecessary if there is one SV caller who always has the best performance. Therefore, the number of long-read sequencing datasets that each SV caller ranks as top one was further compared, as shown in Figure 4.

As shown in Figure 4, the different SV callers rank top one on a certain number of long-read sequencing datasets, and the best performing SV caller can only account for about half of the overall, indicating that the optimal SV caller varies for different long-read sequencing datasets.

### 3.3 Effectiveness of the proposed recommendation method

This section presents the proposed method’s recommendation results regarding RA. Furthermore, it also presents the recommendation performance on real long-read sequencing datasets.

#### 3.3.1 Recommendation accuracy

**FIGURE 5:**
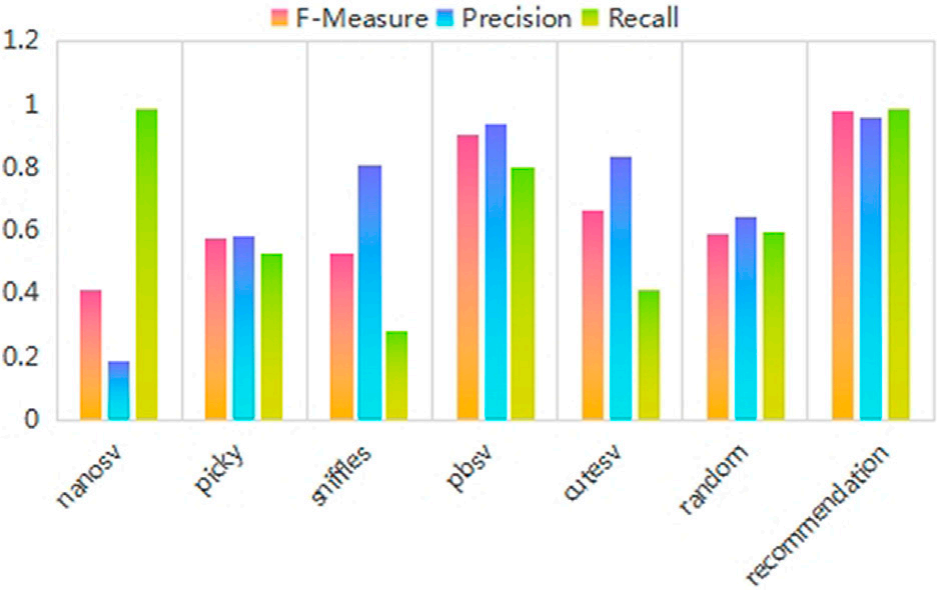
Recommendation accuracy values for three different performance evaluation metrics. The results of the three different performance evaluation metrics are displayed on three different colored bars. The abscissa indicates the five fixed SV callers, a randomly selected SV caller, and the recommended SV caller in order. The ordinate is the recommendation accuracy value.

In this subsection, the recommendation method’s potential usefulness was demonstrated in real practice by comparing the RA values of the recommended SV caller with those of fixed and randomly selected SV callers. The RA describes the performance of the recommended SV caller compared with the best and worst SV caller, which is important in evaluating the usefulness of the SV caller recommendation method. Figure 5 shows the experimental results for the RA value.

As shown in Figure 5, the fixed-pick strategy performs differently for different SV callers. The random-pick strategy has poor performance. The recommended SV caller methods are better and more stable than the random-pick and fixed-pick strategies.

#### 3.3.2 Hypothesis test for recommendation accuracy

**FIGURE 6:**
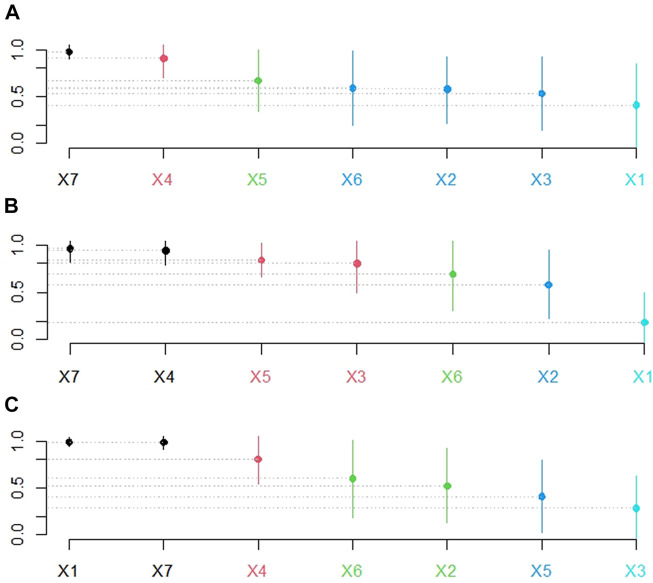
Hypothesis test results of RA values. In **(A)**, **(B)**, and **(C)**, X1-X7 denote the five fixed SV callers, a randomly selected SV caller, and the recommended SV caller in that order. The ordinate represents the recommendation accuracy values. Subfigures **(A)**, **(B)**, and **(C)** represent the recommendation schemes with f-measure, precision, and recall as meta-targets, respectively. The points on the bar graph give the average recommendation accuracy values. The length of the bars reflects the method’s stability. The greater the average recommendation accuracy value, the better the recommendation method; the shorter the bars, the more stable the recommended method. Furthermore, no statistical difference existed between bars of the same color, whereas a significant difference existed between bars of different colors.

The above analysis discovered that the proposed SV caller recommendation method improves the RA values of the random- and fixed-pick strategies. To test whether the improvement is statistically significant, the Scott-Knott effect size difference test (Chakkrit al., 2017) was applied (https://github.com/klainfo/ScottKnottESD), allowing the RA values of different methods to be divided into different groups with non-negligible differences. Figure 6 shows the Scott-Knott effect size difference test results.

Figures 6A, B show that the SV caller recommendation method has superior and more stable performance advantages, whereas Figure 6C shows that in the recommendation scheme with recall as the meta-target, the difference between the recommendation method’s performance and that of fixed with NanoSV is insignificant. Therefore, the following WinDrawLoss analysis experiments were conducted to compare the winners and losers between the different methods.

#### 3.3.3 WinDrawLoss analysis for recommendation accuracy

From the above experiments, the difference in performance between the recommendation method and that of fixed with a specific SV caller is insignificant in the recommendation scheme with recall as the meta-target. Therefore, as presented in Table 2, the WinDrawLoss analysis of the different methods was conducted, showing the number of wins, draws, and losses of different methods on different datasets.

**TABLE 2:** WinDrawLoss Analysis on RA Values.Subtables (A), (B), and (C) represent the recommendation schemes with f-measure, precision, and recall as meta-targets. In each subtable, each line represents the recommended SV caller being compared with the five fixed SV callers and a randomly selected SV caller, and each column represents the number of wins, draws, and losses, respectively.

(A)			
f1score	Win	Draw	Loss
fixed-pick_nanosv	596	134	38
fixed-pick_picky	735	6	27
fixed-pick_sniffles	701	35	32
fixed-pick_pbsv	233	491	44
fixed-pick_cutesv	590	137	41
random-pick	579	157	32

(B)			
Precision	Win	Draw	Loss
fixed-pick_nanosv	727	22	19
fixed-pick_picky	721	16	31
fixed-pick_sniffles	483	189	96
fixed-pick_pbsv	153	550	65
fixed-pick_cutesv	478	249	41
random-pick	533	185	50

(C)			
Recall	Win	Draw	Loss
fixed-pick_nanosv	71	654	43
fixed-pick_picky	669	77	22
fixed-pick_sniffles	736	28	4
fixed-pick_pbsv	385	371	12
fixed-pick_cutesv	672	93	3
random-pick	500	247	21

As presented in Table 2, in each case, the number of wins for the recommended method is much higher than the number of losses. In other words, the proposed SV caller recommendation method has significant advantages over other methods.

#### 3.3.4 Evaluating the recommendation accuracy on real long-read sequencing datasets

To further test the performance of the proposed method on real data, all publicly available triple sequencing data were used with benchmarks. Specifically, the real long-read sequencing data from the well-studied NA12878 individual were used by the Ashkenazim Jewish and Chinese trios to assess the recommendation performance of the proposed SV caller recommendation method (Gong al., 2018; Zook al., 2016). Subreads datasets of the NA12878 individual (HG001), the Ashkenazim Jewish trio son (HG002), the Ashkenazim Jewish trio father (HG003), the Ashkenazim Jewish trio mother (HG004), the Chinese trio son (HG005), the Chinese trio father (HG006), and the Chinese trio mother (HG007) were downloaded from GIAB (https://ftp-trace.ncbi.nlm.nih.gov/giab/ftp/data/).

The experimental results showed that the proposed SV caller recommendation method achieved the RA values of 86.05%, 64.28%, and 95.92% for meta-targets f-measure, precision, and recall, respectively, and the RA remained above 80% on average.

#### 3.3.5 Threats to validity

A possible threat to the validity of this study lies in whether the simulated data used in the empirical study are representative of the broader datasets. Preferring to choose as many real datasets with benchmarks and well-known published simulator pbsim as possible is the primary way for this study to avoid sample bias.

### 3.4 The online recommendation tool

To further facilitate the research community, we incorporated the recommendation method into an online cloud services for genomic data analysis. This cloud system supports user-friendly online Web UI operation, eliminating the heavy work of setting up the running environment. More than 40 bioinformatics analysis tools are integrated on this, with functions covering eight categories including data statistics, data processing, format conversion, data comparison, visualization, table processing, plotting, and advanced tools to meet individual analysis needs.

**FIGURE 7:**
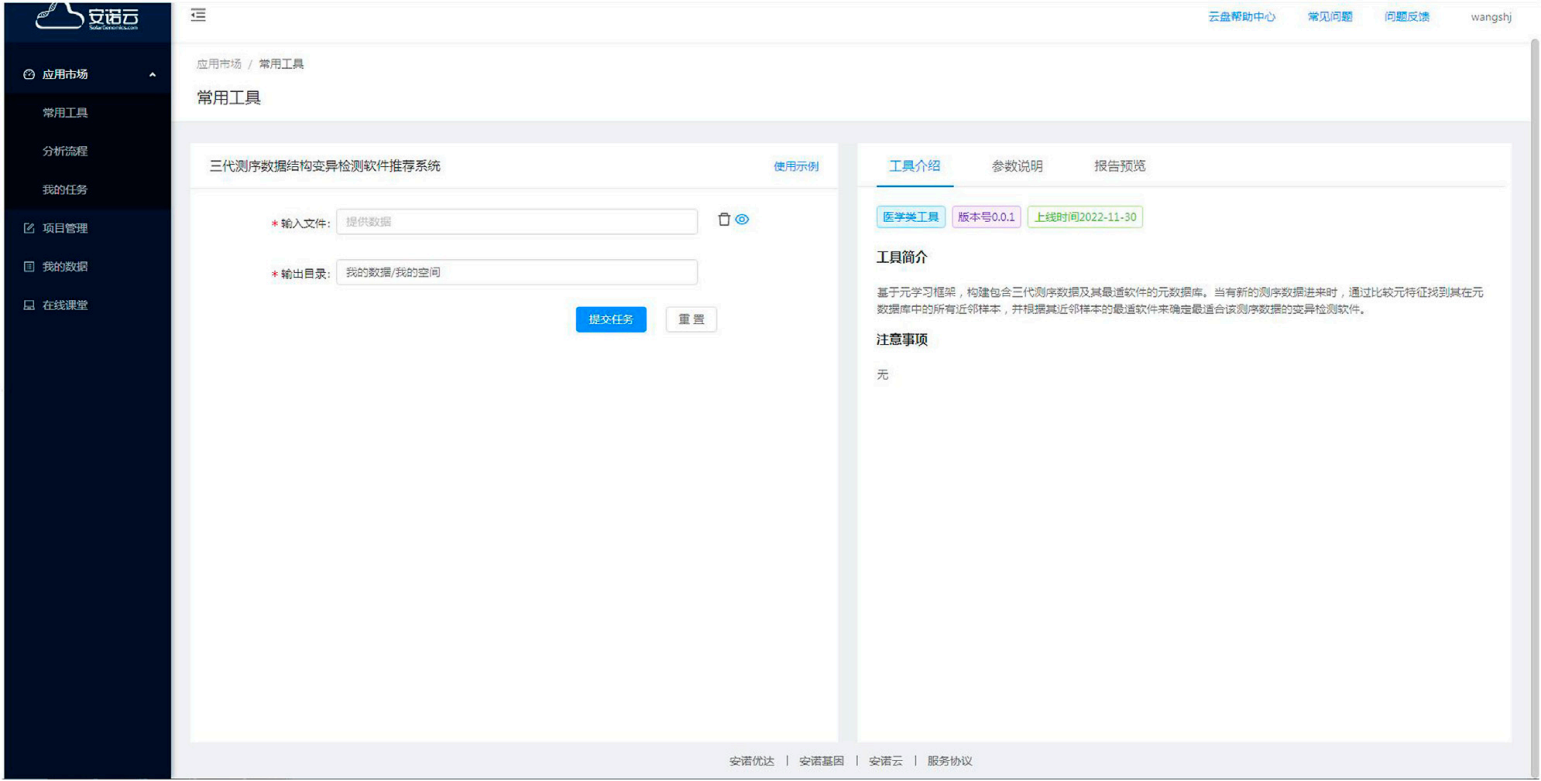
The online recommendation tool input page.

**FIGURE 8:**
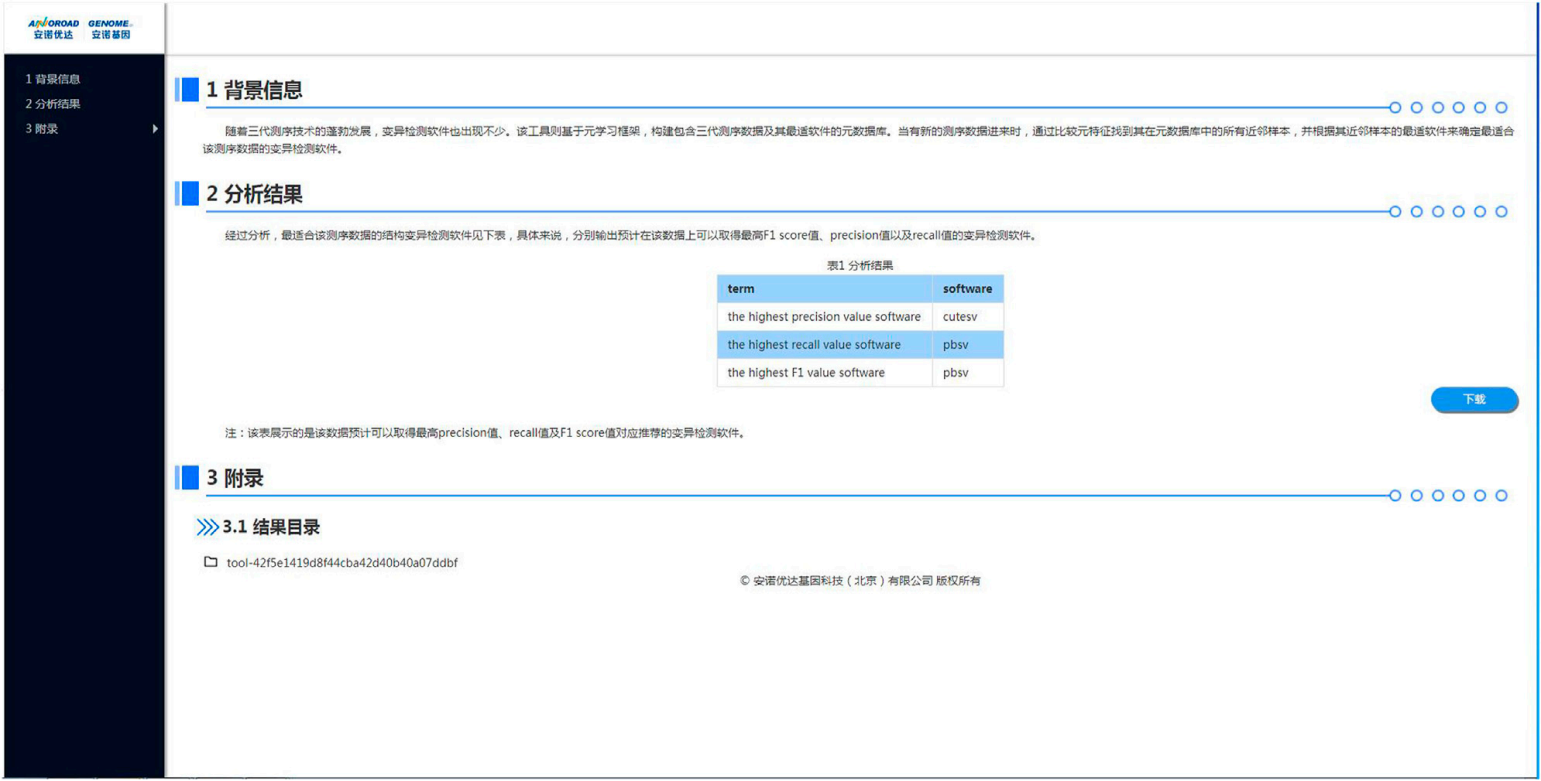
The online recommendation tool output page.

After logged into the cloud system at https://c.solargenomics.com/, users can search for this recommendation tool in the frequently used tools search box. Then, input the fastq file to be analyzed in the file input box and click the submit button, as shown in Figure 7. After the program is finished, you can see the recommended variant calling tool for that data in the task menu, as shown in Figure 8. Currently, the cloud system is collaborated with a PacBio Partner in China, and we are seeking for further collaborations on the cloud systems with English services. In addition, the source code and a pre-trained model is available at https://github.com/hello-json/CallerRecommendation for academic usages only.

## 4 Discussion

Other options for selecting meta-features and the classification algorithm may be available in the proposed recommendation method. Thus, these issues are discussed here. Two widely used classification evaluation metrics, i.e., f-measure and area under the receiver operating characteristic (AUC), were used to evaluate the classification accuracy of the method. Furthermore, a tenfold cross-validation method was used to fully utilize the dataset for the experiments.

**FIGURE 9:**
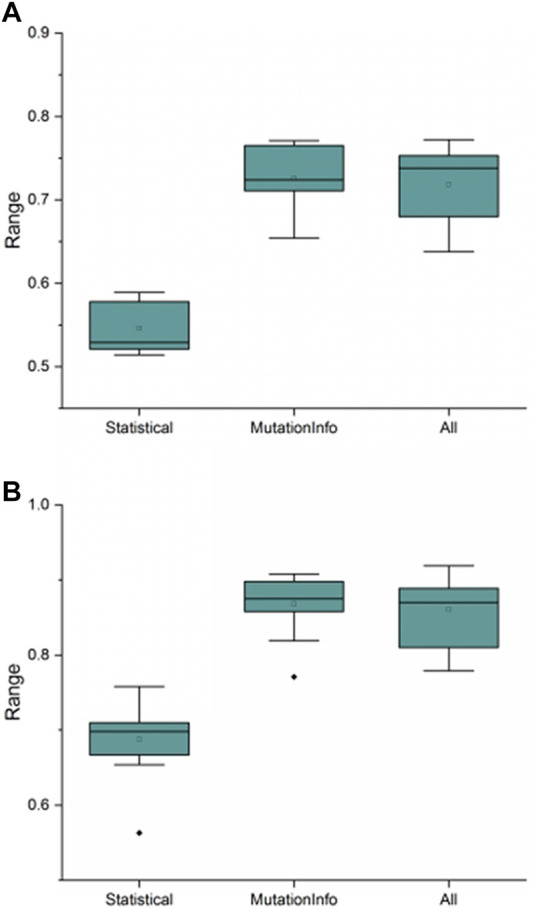
Recommendation performance of different recommendation models constructed with different meta-features. **(A)** and **(B)** show the comparison results of different meta-features on f-measure and AUC performance evaluation metrics, respectively, where “Statistical” denotes the traditional meta-features, “MutationInfo” denotes the variational signature-based meta-features, and “All” denotes the combination of two meta-features. The ordinate indicates the values of the corresponding performance evaluation metrics.

First, the recommendation performance of recommendation models built from meta-features extracted using this study’s variational signature-based meta-feature estimation algorithm was compared to the traditional meta-feature extraction method and a combination of the two meta-feature extraction methods, as shown in Figure 9. As shown in Figure 9, the performance of recommendation methods built with different meta-features varies with the recommendation model built with this study’s variational signature-based meta-features having the best performance and being the most stable.

**FIGURE 10:**
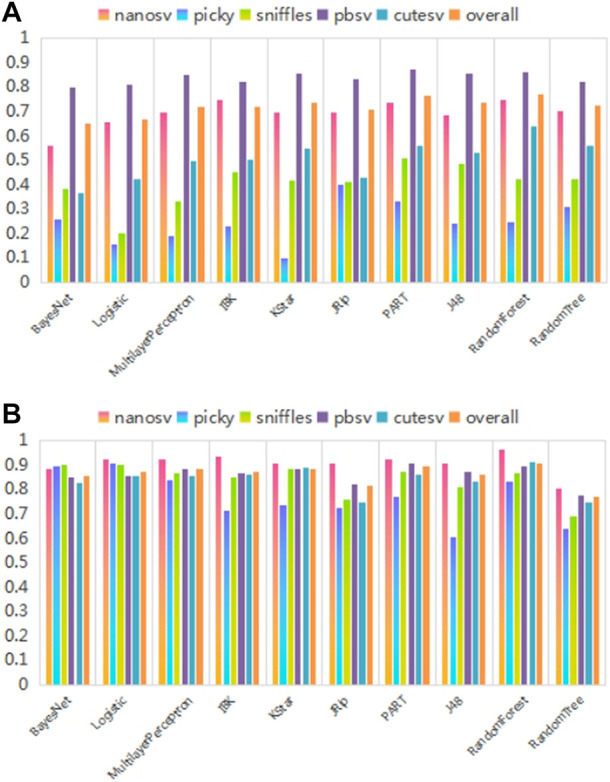
Recommendation performance of recommendation models constructed by different classification algorithms. **(A)** and **(B)** show the comparison results of different classification algorithms on f-measure and AUC performance evaluation metrics, respectively. The abscissa represents ten commonly used multi-classification algorithms based on different principles. The different colored bars for each multi-classification algorithm indicate their recommendation performance for each SV caller and overall. The ordinate is the performance evaluation metric values.

Next, the recommendation performance of recommendation models built using different classification algorithms was compared, as shown in Figure 10. As shown in Figure 10, the recommendation performance of the models built using different multi-classification algorithms differs significantly. Among these, the RandomForest algorithm achieves optimal values for f-measure and AUC performance evaluation metrics. The experimental results are consistent with the theoretical analysis in the method section.

## 5 Conclusion

Many bioinformatics approaches provide powerful algorithmic tools to investigate sequencing data in greater depth. However, quickly selecting the tool that best fits the data form among these state-of-the-art approaches becomes a real and practical level problem. An automatic recommendation method is designed and presented to facilitate the data analysts in selecting the best SV caller based on the sequencing data available. To the best of our knowledge, this is among the first recommendation methods for bioinformatics tools for analyzing sequencing data, and it has the potential to aid the research community.

The proposed method is designed under a meta-learning framework. This is acceptable because identifying the relationships between the data features and the performance of callers is a meta-learning problem. Eight data features distributed at the file and signature levels were selected. The relationship between the features and the optimal caller was then identified through a classification algorithm, RandomForest, and this relationship was used for the caller recommendation. A series of experiments validated the performance and advantages of the automatic recommendation, whatever it takes to recommend the optimal caller with the highest f-measure, precision, or recall. The experimental results also confirmed that different SV callers often fit different samples (sequencing data). The RA was maintained above 80% on average, which was much better than the random-pick and fixed-pick strategies.

## Data Availability

The original contributions presented in the study are included in the article/supplementary material, further inquiries can be directed to the corresponding authors.
